# The Prevalence of and Factors Associated with Sarcopenic Obesity, Sarcopenia, and Obesity Among Korean Adults: Findings from the 2022–2023 Korea National Health and Nutrition Examination Survey

**DOI:** 10.3390/medicina61081424

**Published:** 2025-08-07

**Authors:** Do-Youn Lee

**Affiliations:** College of General Education, Kookmin University, Seoul 02707, Republic of Korea; triptoyoun@kookmin.ac.kr; Tel.: +82-02-910-5540

**Keywords:** sarcopenic obesity, sarcopenia, obesity, prevalence, associated factors

## Abstract

*Background and Objectives*: Sarcopenic obesity, or the coexistence of sarcopenia and obesity, carries an additional load of health risks, including functional decline and metabolic disorders. Despite its increasing importance, data on Korean adults’ prevalence and risk factors are poor. The objective of this study was to estimate the prevalence of sarcopenic obesity, sarcopenia, and obesity to identify factors associated with each condition using the most recent nationally representative data. *Materials and Methods*: This study analyzed data from 4332 adults aged ≥ 40 years who participated in the 2022–2023 Korea National Health and Nutrition Examination Survey (KNHANES). Sarcopenia was defined using the appendicular skeletal muscle index (SMI) via bioelectrical impedance analysis (BIA), and obesity by waist circumference per Korean criteria. Participants were categorized into four body composition groups. Complex sample logistic regression was used to identify factors independently associated with each condition. *Results*: The prevalence rates of sarcopenic obesity, sarcopenia-only, and obesity-only were 1.9%, 14.4%, and 35.5%, respectively. Sarcopenic obesity was significantly more common among older women with low education level, poor subjective health, diabetes, and low HDL-C. They were associated with older age, lower physical activity, lower education level, past smoking, and poor health condition. Obesity was associated with male sex, diabetes, hypertension, dyslipidemia, and moderate-to-poor perceived health. *Conclusions*: Sarcopenic obesity, while less prevalent, is relatively uncommon and represents a high-risk phenotype associated with metabolic and functional deficits. These results highlight the importance of identifying vulnerable subgroups and implementing targeted strategies that address both muscle loss and adiposity in aging Korean adults.

## 1. Introduction

Sarcopenia is an age-related loss of skeletal muscle mass and strength that is one of the most important causes of functional decline and loss of independence in older adults [1]. It often leads to adverse outcomes such as frailty, falls, fractures, and increased mortality [2]. Obesity, meanwhile, has become a global pandemic, with up to 40% of the population now classified as obese by Asian standards in countries like South Korea [3]. Obesity is a major contributor to cardiometabolic diseases including type 2 diabetes, cardiovascular disease, and certain cancers [3]. The coexistence of sarcopenia and obesity—termed sarcopenic obesity—is an emerging public health concern, as it may compound the health risks of each condition [2]. Sarcopenic obesity is associated with greater frailty and disability (e.g., difficulties in mobility and activities of daily living) and a higher risk of chronic diseases than either sarcopenia or obesity alone [2]. Indeed, sarcopenic obesity appears to be a more severe phenotype, synergistically combining low muscle function with excess adiposity to worsen outcomes such as cardiovascular disease and all-cause mortality [2,4]. With global populations aging and obesity prevalence rising, the incidence of sarcopenic obesity is expected to increase rapidly [2,5].

Quantifying the burden of sarcopenia and sarcopenic obesity is essential for public health planning. Globally, over 50 million people were estimated to be sarcopenic in the year 2000, and this number is projected to exceed 200 million by 2040 [6]. The prevalence of sarcopenia increases with age and varies by population and definition: for example, in Asian countries, sarcopenia has been reported in about 5–21% of older men and 4–16% of older women [7,8,9]. In Korea, approximately 18.4% of people aged 70 or older had sarcopenia as of 2017 [10]. Sarcopenic obesity, defined as the concurrence of sarcopenia and obesity, is less common in the general population but can reach appreciable levels in the elderly [11,12]. A recent meta-analysis reported that about 11% of adults aged ≥ 60 worldwide meet criteria for sarcopenic obesity, though estimates vary widely with definitions [12]. Despite its importance, sarcopenic obesity remains understudied, and prior Korean data on its prevalence and risk factors are limited.

This study aimed to determine the prevalence of sarcopenia, sarcopenic obesity, and obesity in Korean adults using nationally representative data from the 2022–2023 Korea National Health and Nutrition Examination Survey (KNHANES). We also sought to identify factors associated with each condition, including sociodemographic characteristics, health-related behaviors, and comorbidities. The results will help emphasize the public health importance of sarcopenia and sarcopenic obesity and inform targeted strategies for prevention and management in aging populations.

## 2. Materials and Methods

### 2.1. Study Design and Participants

This study utilized data from the 2022–2023 Korea National Health and Nutrition Examination Survey (KNHANES), a nationally representative cross-sectional survey conducted by the Korea Centers for Disease Control and Prevention (KCDC). The KNHANES employed a complex, multistage stratified, clustered, and probability sampling design to select participants who represent the non-institutionalized civilian population of Korea.

**Figure 1:**
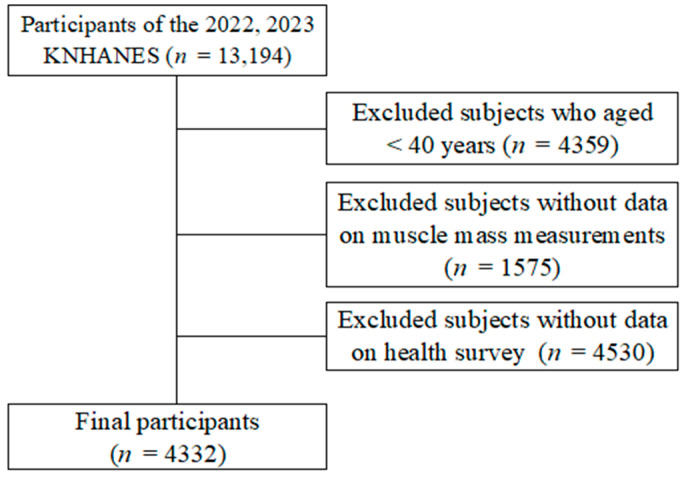
Participant selection process.

Initially, 13,194 participants were included in the KNHANES 2022–2023 datasets. Among these, individuals aged younger than 40 years (*n* = 4359) were excluded, leaving 8835 potential participants aged 40 years and older. Subsequently, individuals with missing data on key variables including muscle mass measurements (*n* = 1575) and health survey data (*n* = 4530) were excluded. Ultimately, a total of 4332 participants aged ≥ 40 years were included in the final analysis (Figure 1).

### 2.2. Definitions of Sarcopenic Obesity, Sarcopenia, and Obesity Sarcopenia

Sarcopenia was defined based exclusively on muscle mass measurement, calculated as the appendicular skeletal muscle mass index (SMI), which was determined by bioelectrical impedance analysis (BIA). SMI was calculated by dividing the appendicular skeletal muscle mass (ASM, kg) by the height squared (m^2^). Participants were classified as sarcopenic if the SMI was <7.0 kg/m^2^ for men and <5.7 kg/m^2^ for women [13].

Obesity was defined based on waist circumference (WC), measured at the midpoint between the lowest rib and the iliac crest. According to the Korean Society for the Study of Obesity guidelines, abdominal obesity was defined as a WC ≥ 90 cm in men and ≥85 cm in women [14].

Participants were classified into four groups based on these criteria: (1) sarcopenic obesity (presence of both sarcopenia and obesity), (2) sarcopenia-alone (presence of sarcopenia without obesity), (3) obesity-alone (presence of obesity without sarcopenia), and (4) normal (absence of both conditions).

#### 2.2.1. Sociodemographic Factors

Participants were categorized into two age groups: middle-aged adults (40–64 years) and older adults (≥65 years). Sex was classified as men or women. Educational level was grouped into four categories: elementary school or lower, middle school, high school, and university or higher. Marital status was classified into two groups: living with a partner or living without a partner. Individual income level was divided into quartiles (Q1—lowest; Q2; Q3; Q4—highest). Residential areas were categorized into urban or rural.

#### 2.2.2. Health-Related Factors

Health-related behavioral and status factors were obtained from KNHANES interviews and examinations. Subjective health status was categorized into three groups: good, moderate, and bad. Perceived stress level was measured on a 4-point Likert scale (from very high to very low) and dichotomized into high stress vs. low stress. Smoking status was categorized as current smoker, former smoker, or never smoker. Alcohol consumption was categorized as current drinker (yes) vs. non-drinker (including never drank or abstained recently) based on survey questions about drinking habits. Physical activity was evaluated using the Global Physical Activity Questionnaire. We focused on aerobic physical activity recommendations: individuals reporting at least 150 min per week of moderate or 75 min of vigorous activity (or an equivalent combination) were classified as engaging in moderate-to-vigorous physical activity (MVPA), while those not meeting this threshold were classified as having low physical activity [15]. For descriptive purposes, we also examined the proportion engaging in strength exercises.

#### 2.2.3. Comorbid Health Conditions

This study considered several comorbid conditions known to be associated with obesity or sarcopenia. Hypertension was defined as a systolic blood pressure ≥ 130 mmHg or diastolic blood pressure ≥ 85 mmHg on the KNHANES examination, or the current use of antihypertensive medication. Diabetes was defined as having a fasting plasma glucose ≥ 126 mg/dL or the current use of antidiabetic medications [16]. Dyslipidemia measures included hypertriglyceridemia (fasting triglycerides ≥ 150 mg/dL or use of lipid-lowering medication) and low HDL cholesterol (HDL < 40 mg/dL in men or <50 mg/dL in women, or on treatment for low HDL) [17].

### 2.3. Statistical Analysis

All statistical analyses were performed using SPSS version 28.0 (IBM Corp., Armonk, NY, USA). Data analyses incorporated the complex sampling design, using sample weights provided by KNHANES. Descriptive statistics are presented as weighted frequencies with percentages. Multiple logistic regression analyses were conducted to calculate odds ratios (ORs) and 95% confidence intervals (CIs) to identify factors associated with sarcopenic obesity, sarcopenia, and obesity. Statistical significance was defined as *p* < 0.05.

## 3. Results

### 3.1. Prevalence of Sarcopenic Obesity, Sarcopenia, and Obesity

**Figure 2:**
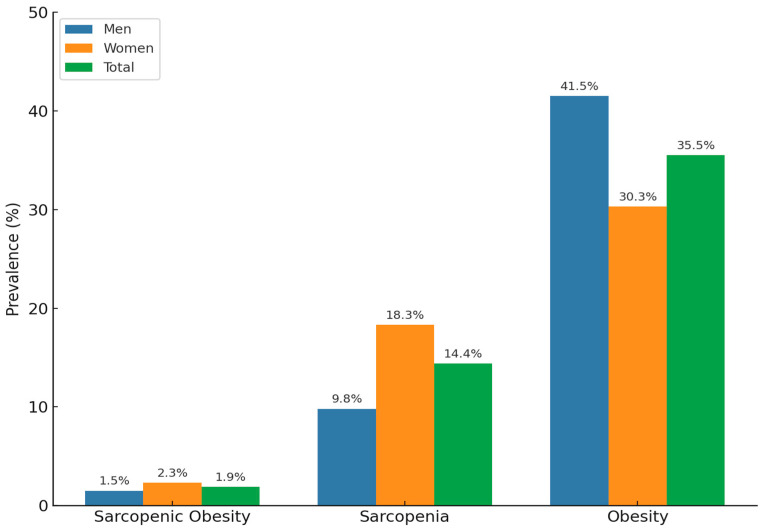
Prevalence of sarcopenia and sarcopenic obesity by sex group.

The overall prevalence rates were 1.9% for sarcopenic obesity, 14.4% for sarcopenia, and 35.5% for obesity. Specifically, sarcopenic obesity was more prevalent among women (2.3%, *n* = 65) compared to men (1.5%, *n* = 36). Similarly, sarcopenia was higher among women (18.3%, *n* = 477) than men (9.8%, *n* = 227). Conversely, obesity prevalence was slightly higher among men (41.5%, *n* = 725) than women (30.3%, *n* = 790) (Figure 2).

### 3.2. Sociodemographic Characteristics of the Study Population

Table 1 presents sociodemographic characteristics by body composition group (sarcopenic obesity, sarcopenia-only, obesity-only, normal). Significant differences in sociodemographic characteristics were observed among the groups (Table 1). Sarcopenic obesity was most prevalent in older adults (89.2%) compared to middle-aged adults (10.8%). Sarcopenia was similarly more common among older adults (44.6%) than middle-aged adults (55.4%), while obesity was predominantly observed in the middle-aged group (68.6%). Women had a higher prevalence of sarcopenic obesity (64.9%) and sarcopenia (68.7%), whereas obesity was more common among men (53.9%). Education level was significantly different across groups; individuals with elementary education had the highest prevalence of sarcopenic obesity (48.6%), whereas those with university-level education were predominantly in the obesity (43.7%) and normal groups (45.4%). Individuals living without a partner had higher proportions in the sarcopenic obesity (35.9%) and sarcopenia groups (19.4%). Lower individual income (Q1) was more frequent in those with sarcopenic obesity (31.5%). Residential area did not differ significantly among the groups.

### 3.3. Health-Related Characteristics of the Study Population

Table 2 compares health-related characteristics across the four groups. Significant differences in health-related characteristics and comorbid conditions were observed among the groups (Table 2). Participants with sarcopenic obesity reported the highest proportion of poor subjective health status (36.7%), followed by those with sarcopenia (22.2%). Higher stress levels were slightly more prevalent in the obesity group (23.3%) compared to other groups. Current smokers were most frequent in the obesity group (17.6%), whereas non-smokers were predominant in the sarcopenia group (71.6%) and sarcopenic obesity group (69.1%). Alcohol consumption was notably higher in the obesity (56.4%) and normal groups (55.7%), compared to the sarcopenic obesity (29.3%) and sarcopenia groups (36.7%). Low physical activity was particularly prevalent in those with sarcopenic obesity (98.7%) and sarcopenia (95.7%). Regarding comorbid conditions, diabetes (62.6%), hypertension (39.4%), high triglycerides (29.2%), and low HDL-C (40.6%) were most prevalent in the sarcopenic obesity group, indicating a strong association with metabolic disturbances (all *p* < 0.001).

### 3.4. Factors Associated with Sarcopenic Obesity, Sarcopenia, and Obesity

Table 3 summarizes the results from the logistic regression analyses. Older age was significantly associated with increased odds of sarcopenic obesity (OR 14.42, 95% CI 7.03–29.57, *p* < 0.001), sarcopenia (OR 2.31, 95% CI 1.77–3.01, *p* < 0.001), and obesity (OR 1.30, 95% CI 1.05–1.61, *p* = 0.016). Men had significantly higher odds of obesity (OR 1.35, 95% CI 1.03–1.77, *p* = 0.032), while sex was not significantly associated with sarcopenic obesity or sarcopenia. Lower education levels were significantly associated with higher odds of sarcopenic obesity (elementary: OR 3.43, 95% CI 1.31–8.97, *p* = 0.012) and sarcopenia (elementary: OR 1.58, 95% CI 1.10–2.29, *p* = 0.015; middle: OR 1.68, 95% CI 1.18–2.40, *p* = 0.004). Poor subjective health status was positively associated with sarcopenic obesity (OR 2.97, 95% CI 1.50–5.85, *p* = 0.002), sarcopenia (OR 2.12, 95% CI 1.51–2.98, *p* < 0.001), and obesity (moderate: OR 1.20, 95% CI 1.00–1.44, *p* = 0.048; bad: OR 1.97, 95% CI 1.54–2.52, *p* < 0.001). Past smoking status showed significantly lower odds of sarcopenia (OR 0.66, 95% CI 0.47–0.92, *p* = 0.014). Alcohol consumption (yes) was associated with lower odds of sarcopenia (OR 0.65, 95% CI 0.47–0.92, *p* = 0.014). Low physical activity was significantly associated with sarcopenia (OR 2.24, 95% CI 1.44–3.48, *p* < 0.001). Among comorbidities, diabetes significantly increased the odds of sarcopenic obesity (OR 1.84, 95% CI 1.14–2.96, *p* = 0.012) and obesity (OR 2.02, 95% CI 1.71–2.35, *p* < 0.001). Hypertension (OR 1.50, 95% CI 1.26–1.78, *p* < 0.001), high triglycerides (OR 1.97, 95% CI 1.60–2.42, *p* < 0.001), and low HDL-C (OR 1.44, 95% CI 1.18–1.76, *p* < 0.001) were significantly associated with obesity, and low HDL-C was also significantly associated with sarcopenic obesity (OR 1.98, 95% CI 1.17–3.36, *p* = 0.011).

## 4. Discussion

This study found that among Korean adults aged 40 years and older, the prevalence rates were 1.9% for sarcopenic obesity, 14.4% for sarcopenia, and 35.5% for obesity. Sarcopenic obesity was predominantly observed in older women with lower education levels, poorer subjective health, and higher prevalence of diabetes and low HDL-C. Sarcopenia was associated significantly with older age, lower education, poor subjective health, past smoking, no alcohol consumption, and low physical activity. Obesity was more common among middle-aged men and was significantly associated with older age, moderate to poor subjective health, diabetes, hypertension, high triglycerides, and low HDL-C. These findings highlight distinct sociodemographic and health-related profiles associated with sarcopenic obesity, sarcopenia, and obesity, indicating the need for targeted prevention and intervention strategies.

The overall characteristics of the study population (*n* = 4332) reflected a balanced distribution by sex (46.0% men, 54.0% women), with a predominance of middle-aged adults (70.8%) and urban residents (85.0%). The prevalence of diabetes (42.9%) and low HDL-C (21.6%) in the total sample was comparable to national estimates for Korean adults aged ≥ 40 years, suggesting that the sample was representative of the broader population. Notably, individuals with sarcopenic obesity were more likely to be older (89.2%), female (64.9%), have lower education levels (48.6% with elementary education), and report poor subjective health (36.7%) and low physical activity (98.7%). These patterns reflect a cluster of socioeconomic disadvantages and metabolic vulnerability in this group.

These findings align with a growing body of literature on the links between sarcopenia, obesity, and metabolic health. Numerous studies confirm that advanced age is the strongest risk factor for sarcopenia. For example, a meta-analysis reported that global sarcopenia prevalence is rising dramatically in the elderly, with adults over 70 having significantly higher odds of sarcopenia than middle-aged adults do [18]. Consistently, sarcopenic obesity is predominantly observed in older populations, reflecting the compounding effects of aging on both muscle loss and fat gain [19,20].

This study observed a higher proportion of women in the sarcopenia groups, which contrasts with some Western studies that report higher sarcopenia prevalence in men [9]. However, other Asian data support our result—for instance, women may be more prone to sarcopenia at older ages due to lower baseline muscle mass and the impact of menopause on muscle metabolism [21]. Previous studies noted that postmenopausal status and low estrogen are associated with increased sarcopenia risk in women, whereas men tend to maintain muscle mass until very advanced ages [22,23]. Overall, the supportive evidence underscores that the result of this study fits within known patterns: aging, physical inactivity, and poor metabolic profile are key correlates of sarcopenia and especially of sarcopenic obesity [18,19,24,25]. The clustering of poor self-rated health with sarcopenia in this study is also expected, as sarcopenia is associated with functional decline and lower quality of life. A recent meta-analysis confirmed that sarcopenic individuals have a significantly reduced health-related quality of life compared to non-sarcopenic peers [26]. This concordance between our findings and the literature adds confidence in the validity of this study’s results.

The role of lifestyle factors is supported by the literature as well. Smoking and heavy alcohol use are generally considered detrimental to muscle health [27,28], whereas our finding of lower sarcopenia odds in moderate drinkers aligns with some reports of a J-shaped relationship between alcohol and muscle outcomes [29]. This may reflect that moderate drinkers in this study had relatively healthier overall profiles or more frequent social engagement, which could indirectly support muscle preservation. Low socioeconomic status also emerged as a risk factor in this study (e.g., lower education associated with sarcopenia), consistent with previous findings. Swan et al. (2021) observed that older adults with only primary education had a significantly higher prevalence of sarcopenia than those with college education, likely reflecting differences in lifetime nutrition and health behaviors [30]. Similarly, an Iranian study found sarcopenia rates were highest in low-income elderly and lowest in high-income groups, pointing to socioeconomic disparities in muscle health [30].

There is robust evidence that low physical activity contributes to sarcopenia. A systematic review found older adults with high levels of physical activity had a substantially lower prevalence of sarcopenia, whereas those with sedentary lifestyles were at elevated risk [25]. This supports our observation that moderate-to-vigorous activity was protective against sarcopenia. Encouragingly, resistance exercise is known to improve muscle mass and function in older adults, underscoring physical inactivity as a modifiable risk factor for sarcopenia [25].

This study noted a strong association between sarcopenic obesity and metabolic derangements (high prevalence of type 2 diabetes, low HDL cholesterol, high triglycerides), which is in line with prior research. Individuals with the dual burden of low muscle and high fat have been shown to exhibit worse insulin resistance and lipid profiles than those with obesity or sarcopenia alone [24]. Habib et al. reported that obese men with sarcopenia had higher insulin levels, HOMA-IR, and triglycerides and lower HDL-C compared to non-sarcopenic obese controls [24]. Epidemiologically, diabetes is often associated with sarcopenia: a 2021 meta-analysis found that people with diabetes had over twice the odds of sarcopenia compared to non-diabetics [24]. Hyperglycemia, insulin resistance, and chronic inflammation in diabetes likely contribute to accelerated muscle loss [31]. In this study, diabetes was significantly linked to sarcopenic obesity, reinforcing the interplay between impaired glucose metabolism and the sarcopenia-obesity phenotype.

Despite the general agreement, some aspects of this study’s findings diverge from other reports, highlighting areas of controversy. We did not find a significant association between current smoking and sarcopenia, yet prior studies suggest smoking is a risk factor for muscle loss. Locquet et al. (2021) reported that older adults who smoke had a ~2.7-fold higher risk of developing severe sarcopenia over 5 years compared to never-smokers [32]. The lack of association in this cross-sectional analysis could be due to survivorship bias (frail smokers may not survive or remain in the cohort) or differences in smoking intensity and cohort characteristics.

This study finds that moderate alcohol use was associated with lower sarcopenia prevalence, which is somewhat counterintuitive, as excessive alcohol is known to damage skeletal muscle. A recent study actually found a sex-specific effect: moderate drinking was linked to lower odds of sarcopenia in women, whereas in men, the benefit was not observed, possibly due to different drinking patterns [33]. In general, chronic heavy alcohol intake clearly has negative effects on muscle (via oxidative stress and protein synthesis inhibition), and any observed protective association likely applies only to light intake. The divergent literature on lifestyle factors indicates that dose and context are critical—mild exposures might have neutral or even beneficial effects, whereas heavier exposures are uniformly detrimental. In summary, most contradictions can be reconciled by differences in definitions, population demographics, and exposure levels. These discrepancies underscore the need for careful interpretation and further research. For instance, whether a mild “obesity paradox” exists for certain outcomes in sarcopenic older adults remains debated, and longitudinal studies are needed to clarify the long-term impact of adiposity in those with low muscle. Similarly, standardized criteria for sarcopenia that include muscle function (strength or performance) may yield different associations with metabolic diseases than criteria based on muscle mass alone, as seen across studies.

Another point of discrepancy is whether sarcopenia synergistically worsens metabolic risk beyond obesity alone. We found that sarcopenic obesity carried especially high odds of dyslipidemia and diabetes, but an Australian longitudinal study (CHAMP) reported that sarcopenic obesity did not confer a significantly greater 5-year risk of metabolic syndrome or insulin resistance than obesity without sarcopenia [34]. The authors used strict criteria including low gait speed, which might select a more severe sarcopenia subset; by contrast, the sarcopenia definition (based on low muscle mass) of this study could include metabolically healthier individuals. This difference in findings suggests that the interaction of low muscle and obesity on cardiometabolic outcomes may depend on how sarcopenia is defined (mass vs. function) and the population studied (men in the CHAMP study vs. a general Korean population in this study).

Sarcopenia and obesity are interconnected through several biological pathways. Aging itself promotes a loss of muscle mass and strength while favoring fat accumulation due to hormonal changes and reduced activity [19]. Inflammatory crosstalk between adipose and muscle tissue appears central: excess adipose tissue in obesity releases pro-inflammatory cytokines (e.g., TNF-α, IL-6) and leads to fat infiltration into muscle (myosteatosis), which in turn accelerates muscle protein breakdown and mitochondrial dysfunction. Insulin resistance is a key mechanistic link—skeletal muscle is the major insulin-sensitive tissue, and with sarcopenia, there is less muscle to uptake glucose, exacerbating insulin resistance. Conversely, insulin-resistant states (such as in abdominal obesity or type 2 diabetes) impair muscle protein synthesis and anabolic signaling, hastening muscle atrophy [20,31]. Physical inactivity further compounds these processes; the disuse of muscle leads to atrophy and strength loss, and a sedentary lifestyle in middle-aged and older adults facilitates fat gain, creating a vicious cycle. Additionally, age-related declines in anabolic hormones (growth hormone, IGF-1, testosterone) and, in women, the loss of estrogen after menopause contribute to reduced muscle regeneration and increased fat mass [19,35]. The net result of these mechanisms is a self-perpetuating cycle in which obesity-driven inflammation and insulin resistance accelerate muscle catabolism, and low muscle mass in turn worsens metabolic dysfunction—an unhealthy synergy that has been termed “sarcopenic obesity.”

This study used a large, nationally representative dataset (KNHANES 2022–2023) with standardized measurements and AWGS-based BIA criteria to define sarcopenia. By distinguishing four body composition phenotypes (normal, obesity-only, sarcopenia-only, sarcopenic obesity), the analysis clarified the specific risks associated with sarcopenic obesity. Multivariable adjustment for a broad range of sociodemographic, lifestyle, and clinical variables further strengthens the findings. Despite its strengths, this study has several limitations. First, its cross-sectional design precludes any inference of causality. While associations between sarcopenia, obesity, and metabolic diseases were identified, the directionality of these relationships cannot be determined. Second, sarcopenia was defined solely based on low appendicular muscle mass using BIA, without incorporating measures of physical performance (e.g., gait speed). As a result, some individuals may have been misclassified, and the diagnosis may not fully reflect functional impairment, which is an important component of sarcopenia in recent consensus definitions. Third, BIA, while practical in large-scale surveys, is less accurate than dual-energy X-ray absorptiometry (DXA) and may overestimate or underestimate muscle and fat mass depending on hydration status and other factors. Fourth, several key health behavior variables—such as physical activity, smoking, and alcohol intake—were collected through self-report, introducing the possibility of recall or reporting bias. Fifth, dietary intake and inflammatory biomarkers, which are known to influence muscle health and metabolic outcomes, were not analyzed in this study, limiting interpretation of potential mediating factors. Sixth, this study did not incorporate information on menopausal status, which limited our ability to examine the influence of hormonal changes such as estrogen decline on sarcopenia in women. Given the known association between postmenopausal status and muscle loss, future research should include hormonal or reproductive health variables to clarify sex-specific mechanisms.

## 5. Conclusions

This study identified the prevalence and associated factors of sarcopenia, obesity, and sarcopenic obesity among Korean adults using recent nationally representative data from KNHANES 2022–2023. Sarcopenic obesity, though relatively rare, was most prevalent among older women with low education, poor subjective health, diabetes, and dyslipidemia, highlighting its clinical and public health significance. Sarcopenia and obesity each demonstrated distinct sociodemographic and behavioral risk profiles, with aging, physical inactivity, and poor metabolic health emerging as shared determinants. The findings reinforce the notion that sarcopenic obesity represents a synergistically detrimental phenotype, combining the adverse effects of both low muscle mass and excess adiposity. Given the aging population and increasing obesity burden, these results underscore the need for targeted interventions that address both muscle loss and metabolic dysfunction, particularly among vulnerable subgroups.

## Figures and Tables

**Table 1:** Sociodemographic characteristics of the study population.

Factors	Categories	Total (*n* = 4332)	Sarcopenic Obesity (*n* = 101)	Sarcopenia (*n* = 704)	Obesity (*n* = 1515)	Normal (*n* = 2012)	*p*
Age	Middle	70.8	10.8	55.4	68.6	79.5	<0.001
	Older	29.2	89.2	44.6	31.4	20.5	
Sex	Male	46.0	35.1	31.3	53.9	45.0	<0.001
	Female	54.0	64.9	68.7	46.1	55.0	
Education level	elementary	11.8	48.6	19.4	12.8	7.4	<0.001
	middle	10.1	21.1	14.5	11.6	7.31	
	high	32.8	18.4	32	32	34.2	
	university	45.4	11.8	34.1	43.7	51.3	
Marital status	with	84.6	64.1	80.6	83.0	87.8	<0.001
	without	15.4	35.9	19.4	17.0	12.2	
Individual income	Q1 (Lowest)	20.2	31.5	21.2	21.0	18.9	0.004
	Q2	24.6	19.4	26.1	24.0	24.7	
	Q3	26.7	26.2	22.0	29.7	26.0	
	Q4 (Highest)	28.5	22.9	30.7	25.2	30.4	
Residential area	Urban	85.0	85.0	85.7	84.3	85.3	0.820
	Rural	15.0	15.0	14.3	15.7	14.7	

**Table 2:** Health-related characteristics of the study population.

Factors	Categories	Total (*n* = 4332)	Sarcopenic Obesity (*n* = 101)	Sarcopenia (*n* = 704)	Obesity (*n* = 1515)	Normal (*n* = 2012)	*p*
Subjective health status	Good	34.7	21.3	29.0	30.4	40.2	<0.001
	Moderate	48.8	42.0	48.8	49.3	48.8	
	Bad	16.4	36.7	22.2	20.3	11.0	
Stress level	High	20.7	20.0	18.2	23.3	19.6	0.038
	Low	79.3	80.0	81.8	76.7	80.4	
Smoking status	Current	15.5	9.0	13.1	17.6	15.0	<0.001
	Past	25.6	21.9	15.3	29.8	25.7	
	Non	58.9	69.1	71.6	52.6	59.3	
Alcohol status	Yes	52.7	29.3	36.7	56.4	55.7	<0.001
	No	47.3	70.7	63.3	43.6	44.3	
Physical activity	Low	89.9	98.7	95.7	90.6	87.4	<0.001
	Moderate-vigorous	10.1	1.3	4.3	9.4	12.6	
Comorbidity conditions							
Diabetes		42.9	62.6	39.0	56.3	33.3	<0.001
Hypertension		29.5	39.4	28.4	37.1	23.9	<0.001
High triglyceride		25.9	29.2	17.0	38.0	19.5	<0.001
Low HDL-C		21.6	40.6	16.1	29.6	16.5	<0.001

**Table 3:** Logistic regression analysis for factors associated with sarcopenic obesity, sarcopenia, and obesity.

Categories		Sarcopenic Obesity		Sarcopenia		Obesity	
		OR (95% CI)	*p*	OR (95% CI)	*p*	OR (95% CI)	*p*
Age	Middle	1 (reference)		1 (reference)		1 (reference)	
	Older	14.42 (7.03–29.57)	<0.001	2.31 (1.77–3.01)	<0.001	1.30 (1.05–1.61)	0.016
Sex	Male	1.15 (0.56–2.37)	0.697	0.78 (0.57–1.06)	0.112	1.35 (1.03–1.77)	0.032
	Female	1 (reference)		1 (reference)		1 (reference)	
Education level	Elementary	3.43 (1.31–8.97)	0.012	1.58 (1.10–2.29)	0.015	1.33 (0.98–1.81)	0.069
	Middle	2.53 (0.94–6.78)	0.065	1.68 (1.18–2.40)	0.004	1.35 (1.02–1.79)	0.038
	High	1.24 (0.56–2.74)	0.597	1.13 (0.88–1.46)	0.338	0.94 (0.78–1.12)	0.488
	University	1 (reference)		1 (reference)		1 (reference)	
Marital status	With	0.70 (0.42–1.16)	0.161	0.98 (0.75–1.28)	0.866	0.85 (0.68–1.06)	0.153
	Without	1 (reference)		1 (reference)		1 (reference)	
Individual income	Q1 (Lowest)	1.37 (0.71–2.65)	0.348	0.91 (0.69–1.21)	0.521	1.08 (0.85–1.38)	0.511
	Q2	0.87 (0.43–1.77)	0.698	0.97 (0.72–1.29)	0.811	1.04 (0.81–1.34)	0.746
	Q3	1.20 (0.59–2.46)	0.614	0.80 (0.59–1.08)	0.144	1.29 (1.03–1.60)	0.026
	Q4 (Highest)	1 (reference)		1 (reference)		1 (reference)	
Residential area	Urban	1.71 (0.77–3.82)	0.186	1.26 (0.96–1.66)	0.093	1.16 (0.93–1.44)	0.188
	Rural	1 (reference)		1 (reference)		1 (reference)	
Subjective health status	Good	1 (reference)		1 (reference)		1 (reference)	
	Moderate	1.20 (0.64–2.24)	0.565	1.24 (0.99–1.54)	0.056	1.20 (1.00–1.44)	0.048
	Bad	2.97 (1.50–5.85)	0.002	2.12 (1.51–2.98)	<0.001	1.97 (1.54–2.52)	<0.001
Stress level	High	1.43 (0.80–2.57)	0.225	0.95 (0.72–1.26)	0.734	1.20 (0.97–1.47)	0.086
	Low	1 (reference)		1 (reference)		1 (reference)	
Smoking status	Current	1.21 (0.82–1.78)	0.327	1.21 (0.82–1.78)	0.327	0.81 (0.59–1.12)	0.210
	Past	0.80 (0.41–1.57)	0.796	0.66 (0.47–0.92)	0.014	0.94 (0.70–1.25)	0.645
	Non	1 (reference)		1 (reference)		1 (reference)	
Alcohol status	Yes	0.83 (0.47–1.45)	0.511	0.65 (0.47–0.92)	0.014	1.06 (0.89–1.26)	0.520
	No	1 (reference)		1 (reference)		1 (reference)	
Physical activity	Low	5.30 (0.70–40.01)	0.105	2.24 (1.44–3.48)	<0.001	1.29 (0.98–1.71)	0.070
	Moderate-vigorous	1 (reference)		1 (reference)		1 (reference)	
Comorbidity conditions							
Diabetes		1.84 (1.14–2.96)	0.012	1.15 (0.93–1.42)	0.202	2.02 (1.71–2.35)	<0.001
Hypertension		1.11 (0.66–1.86)	0.703	1.07 (0.85–1.35)	0.564	1.50 (1.26–1.78)	<0.001
High triglyceride		1.49 (0.87–2.56)	0.146	1.01 (0.76–1.36)	0.918	1.97 (1.60–2.42)	<0.001
Low HDL-C		1.98 (1.17–3.36)	0.011	0.72 (0.54–0.96)	0.028	1.44 (1.18–1.76)	<0.001

## Data Availability

All data were anonymized and can be downloaded from the website (https://knhanes.kdca.go.kr/knhanes, accessed on 8 March 2025).
